# Exploring the frontier of dental education: a cross-sectional study of VR simulation and manikin-based training at Ziauddin university

**DOI:** 10.1186/s12909-025-07221-8

**Published:** 2025-05-12

**Authors:** Ammarah Muhammad Nauman, Sidra Mohiuddin, Maryam Panhwar, Yousra Altaf

**Affiliations:** Department of Community and Preventive Dentistry, Ziauddin University, Karachi, Pakistan

**Keywords:** Dental education, Virtual reality in dentistry, Manikin-based training, Dental Phantom head, Dental simulation, Preclinical training tools, Immersive learning technologies

## Abstract

**Background:**

Dental education blends theoretical concepts with practical tasks, where preclinical simulations using manikins have long been integral. However, the limitations of manikin-based training, such as cost, material restrictions, and inter-rater reliability concerns, have led to the integration of emerging technologies like Virtual Reality (VR) to enhance learning. VR provides an immersive environment to practice clinical skills, offering potential flexibility, engagement, and tactile learning advantages. This study compares dental students’ perceptions of VR and manikin training at Ziauddin University, Karachi.

**Methodology:**

This cross-sectional study was conducted at Ziauddin University College of Dentistry, Karachi, Pakistan, involving 229 dental students enrolled in various levels of the BDS program. A structured questionnaire assessed students’ experiences and perceptions of VR simulation and manikin training. Data was analyzed using SPSS version 22, with descriptive statistics and independent T-tests to evaluate differences in perception across student groups.

**Results:**

Both VR and manikin training were effective in improving learning. Manikins were preferred for realistic clinical scenarios, while VR was favored for engagement and tactile learning. 68.6% of students found both methods equally useful for reinforcing knowledge, 77.7% felt more confident after manikin training, and 97.4% found VR effective for understanding tooth textures.

**Conclusion:**

This study highlights the complementary strengths of VR and manikin-based training in dental education. Both methods should be integrated to provide a more effective and well-rounded learning experience. Further research is needed to explore VR’s cost-effectiveness and use in resource-limited settings.

**Supplementary Information:**

The online version contains supplementary material available at 10.1186/s12909-025-07221-8.

## Introduction

### Background

Dental education integrates theoretical concepts, practical lab tasks, and clinical drills, distinguishing it from other health fields [1, 2]. Undergraduate students must become proficient in operating procedures to provide safe and effective clinical care [3]. Preclinical learning simulations ensure safe treatment, focusing on fine motor abilities and hand-foot and eye coordination. Simulations provide a secure learning environment for instruction and evaluation [3–5]. Preclinical training often uses extracted and plastic teeth mounted on manikins, a method used in dental schools for decades. However, this method incurs costs, limits student rehearsing, requires qualified supervisors, and may cause inter-rater reliability issues [3, 5, 6]. 

Advancements in Information Technology (IT) have led to virtual reality (VR) integration in oral health education, with the vision of improving dentistry and enabling independent learning and skill development [2, 7]. VR generates an immersive virtual setting using software that enables users to learn from experience [8]. It effectively enhances the teaching and learning experience by enabling facilitators to conduct challenging, impossible learning tasks during traditional sessions [9]. 

Recent studies highlight the increasing interest in using VR technology in dental education. A systemic review suggests that VR greatly enhances dental students’ academic understanding and practical skills. These systems provide regular training and real-time experience in a virtual environment and bypass environmental constraints. While its applications vary widely, it has the potential to significantly alter the education of competent dental students and enhance traditional teaching techniques [1]. While VR simulation offers advantages, it’s not a panpharmacon; instead, it’s a tool for specific learning goals that must be integrated into the curriculum and pedagogy of an institution to be used effectively [8, 10]. However, there remains a gap in comprehensive comparative analyses of student perceptions towards VR simulations versus traditional manikins, particularly in underprivileged regions or institutions where such technologies are newly introduced.

The inspiration for this research stems from the introduction of Virtual Reality Dental Simulators, known as Virteasy Dental, at Ziauddin University (ZU) in Pakistan, marking the first time this technology has been introduced in the country for dental education and representing a substantial shift from traditional teaching methods. As this innovative technology debuted in the region’s dental education sector, there was a conspicuous gap in empirical research concerning its impact. This absence of regional studies underscores the critical need for a comprehensive investigation to understand how VR simulations, as a novel educational tool, compare with traditional dental manikins in enhancing students’ learning experiences. The research aims to contribute to the knowledge of the benefits, limitations, and educational outcomes of VR technology, guiding future pedagogical strategies and technological integrations in dental education.

Moreover, this study acknowledges the global trend towards adopting immersive technologies in education and positions itself as a vital exploration of such innovations within a Pakistani context. It fills a critical knowledge gap and sets the groundwork for future advancements in dental education methodologies in Pakistan and similar settings where such technology is emerging.

This study aims to explore and compare dental students’ perceptions of VR simulation with traditional dental manikins as teaching tools at Ziauddin University.

### Objective

1. To compare dental students’ perceptions of Virtual Reality simulation with traditional dental manikins.
2. To evaluate the impact of VR simulation on students’ confidence and perceived preparedness for clinical practice.

## Methodology

This cross-sectional study was conducted at Ziauddin University-College of Dentistry in Karachi, Pakistan. A total of *n* = 229 responses were received from dental students enrolled at various levels of their BDS program at ZU, specifically those in the second, third, and fourth years and recent house officers. Students who did not consent to the study or were absent during data collection were excluded.

The training included the objectives of Operative Dentistry. Both VR and manikin training were integrated into the preclinical curriculum for students in the BDS program at ZU and conducted in parallel sessions. Students received equal practice hours for each teaching modality. Each session was conducted in small groups. First, trained faculty members demonstrated all of the tasks, after which the students performed under the demonstrator’s supervision.

The immersive VR training was conducted using the Virteasy VR system, which integrated haptic feedback and a 3D simulated environment. The system simulated dental procedures such as cavity preparation and endodontic access opening, with students actively participating by performing tasks using the VR handpiece. The training was conducted in weekly sessions over seven weeks, with the first week focusing on system and instrument familiarization exercises, followed by Class I, II, III, IV & V cavity preparations and endodontic access opening exercises.

For manikin training, moderate-fidelity manikins were employed to practice the same dental procedures under both direct and indirect vision. Like VR training, manikin sessions followed a structured schedule, with weekly exercises focusing on manikin unit familiarization and instrument identification and use, followed by cavity preparations and endodontic access opening. Manikins were also used to train students in placing rubber dams for isolation and matrix band assembly for Class II fillings.

Task completion for VR training was marked using the built-in evaluation system, which provided feedback for each task. In contrast, the facilitator evaluated the performance in manikin training.

The clinical group (fourth-year students and house officers) applied these skills to real patients after completing training with VR and manikins. In contrast, the preclinical group (second and third-year students) practiced only within simulated environments.

The computer software OpenEpi was used to calculate the sample size by taking the reference values of 85% of participants who supported using Virtual Reality Dental Simulators [3]. This sample size was calculated with a 95% Confidence Interval, 80% Power of test, and 5% margin of error. Hence, a total sample size of 196 (n) was obtained. Adding to it a 10% non-response rate, a total sample of 216 was achieved. Therefore, the sample size of 216 was rounded to 220. Non-probability convenience sampling was employed to recruit participants from the defined population, ensuring a practical approach to data collection while capturing diverse insights.

A structured questionnaire was developed from previous literature findings to assess students’ perceptions and experiences with VR simulation and traditional manikin training. (The English language version of the questionnaire is attached as a supplementary file). It comprised five sections: Section one included three questions about socio-demographics: students’ gender, age, and year of study in the dental program. Sections two and three had nine questions each about the experience with dental manikins and virtual reality training, respectively. The responses were based on a 5-point Likert scale ranging from completely agree to completely disagree [10]. Section four had ten questions related to comparing the training experience with both learning modalities, giving insight into user preference [1, 7]. Section five had five open-ended questions to solicit feedback on using conventional phantom heads and restrictions or recommendations on applying VR to dental skill training. Three experts reviewed the questionnaire for content validity and assessed its relevance. Later, a pilot test was conducted to determine clarity, understandability, and reliability among target users. The internal consistency of the questionnaire was assessed using Cronbach’s alpha, yielding a value of 0.89, indicating good reliability.

Ethical approval (reference code: 8830724ANCPD) for the study was obtained from the Ethics Review Committee (ERC) at Ziauddin University. Informed consent was secured from all participants before data collection. The questionnaires were distributed electronically using Google Docs. Reminders were sent periodically during the one-month data collection period to encourage participation and ensure a high response rate. All respondents were assured anonymity and confidentiality.

Data analysis was conducted using SPSS version 22. Since the respondents were from preclinical and clinical groups, we analysed the responses separately for each cohort to compare perceptions based on clinical exposure. Descriptive statistics were reported as frequencies and percentages. Independent T-tests were applied to investigate the difference in perception between preclinical and clinical students regarding Dental Manikin and Virtual Reality training methods. The level of significance was considered to be less than 0.05.

## Results

Out of a total of *n* = 229, the gender-wise distribution showed the predominance of 80.3% females (*n* = 184) over 19.7% males (*n* = 45). Study participants’ ages ranged from 19 to 27 years, with a mean of 22.79 and a standard deviation of 1.52. They were enrolled in different levels of the Dental Program and divided into two groups: 39.3% (*n* = 90) participants in the preclinical phase (69 in the 2nd year and 21 in the 3rd year) and 60.7% (*n* = 139) participants in the clinical phase (52 in the 4th year and 87 in the House job).

There is a statistically significant difference in perception of dental manikin training among pre-clinical and clinical students (*p* < 0.001, 95% CI -2.93, 0.35). (Table 1)

**Table 1:** Perception of preclinical versus clinical students regarding dental manikin training

Variable	Mean (SD) Preclinical versus clinical students	Mean difference (95% CI)	t-statistic (df)	*p*-value
Dental manikin training	34.67(7.53) 35.96(5.10)	-1.29 (-2.93, 0.35)	-1.55 (227)	< 0.001*

Independent t-test, statistically significant*

However, the difference in perception of virtual reality training among pre-clinical and clinical students is statistically insignificant (*p* = 0.006, 95% CI -1.10, 2.51). (Table 2)

**Table 2:** Perception of preclinical versus clinical students regarding virtual training

Variable	Mean (SD) Preclinical versus clinical students	Mean difference (95% CI)	t-statistic (df)	*p*-value
Virtual reality training	34.37(7.55) 33.66(6.23)	0.70 (-1.10, 2.51)	0.77 (227)	0.006*

Independent t-test, statistically insignificant*

The comparison between virtual reality and dental manikin experiences among preclinical and clinical students is described as the majority of the students, 68.6% (*n* = 157), stated that both training methods are equally helpful in consolidating theoretical knowledge. About 69% (*n* = 158) of the students reported improved learning proficiency after using dental manikins and virtual reality training. When evaluating the realism of clinical scenarios, 79.9% (*n* = 183) of the students indicated a preference for dental manikins. Likewise, 77.3% (*n* = 177) expressed that training with manikins better equipped them for actual dental procedures, highlighting its importance in developing practical skills.

**Fig. 1:**
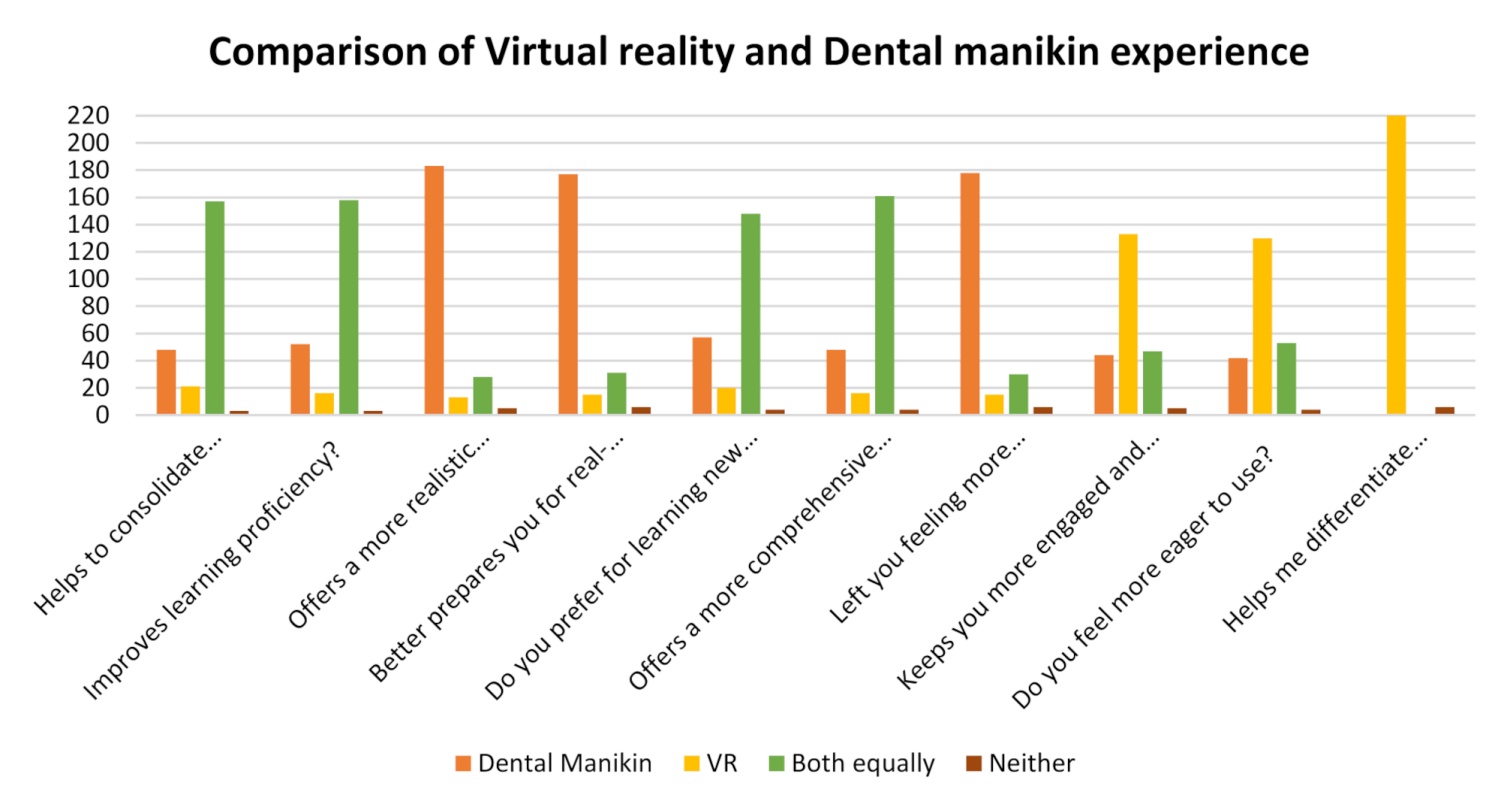
Comparison of virtual reality and dental manikin experience among preclinical and clinical students

Regarding preference for learning new clinical skills, 64.6% (*n* = 148) of students stated that both methods were equally effective. 70.3% (*n* = 161) of students stated that both methods provided a more comprehensive understanding of dental procedures. 77.7% (*n* = 178) of students felt more confident after manikin training. VR was seen as more engaging and motivating, with 58.1% (*n* = 133) of students favoring it for keeping their interest, while 56.8% (*n* = 130) desired to use VR again. Impressively, 97.4% (*n* = 223) of students agreed that VR was effective in helping them distinguish between the texture and hardness of enamel and dentine, underscoring its unique advantages in improving tactile comprehension. (Fig. 1)

## Discussion

An essential component of undergraduate dental education is simulation to help bridge the gap between theoretical knowledge and practical application [1, 2, 11]. This study evaluated dental students’ perception of Virtual Reality (VR) and manikins as teaching tools. Based on the results, clinical students preferred manikins slightly more, but there was no significant difference in VR perceptions between preclinical and clinical groups. Most students found both methods equally helpful for reinforcing knowledge and improving their learning. Manikins were favored for simulating realistic clinical scenarios and boosting confidence. VR, in addition to helping them better understand the texture and hardness of different layers of teeth, fostered more engagement and sensory learning.

Since 1894, traditional dental manikins have been an essential part of dental education [2, 11–13]. Following that, they have developed considerably, adding necessary elements like water spray and dental handpieces to give learners a more realistic environment for diagnosing and treating patients [14]. While highly rated for simulating actual clinical practice, students still find them inadequate due to a lack of feedback as a self-assessment tool and an inability to replicate physical features that affect procedures, such as a moving tongue [15]. This is consistent with our findings, as one participant stated, “*A major issue I faced when transitioning from training on phantom head to working on an actual patient was protecting tongue during crown preparation so that my instruments do not harm the patient*.” Another participant stated, “*It does not provide critical feedback. Precision is a skill I feel I can practice better in VR rather than manikins*.” Furthermore, manikin training necessitates constant material costs and few possibilities for students to practice repeatedly [3]. As one participant stated, “*Artificial teeth are delicate and can easily be ruined when practicing cavity and crown preparation*,* so we have to get new every time*.” Despite these drawbacks, the manikin was highly valued for critical tasks in Class II fillings and rubber dam placement. One participant stated, “*Phantom head helped a lot in correct placement of retainer and wedge in class II preparations and practice rubber dam*.”

Studies have highlighted the importance of digital tools and technology-enhanced simulations, such as virtual reality, in health professional education [10, 16, 17]. Although virtual reality technology has shown to be a promising tool in dental education, it also faces some challenges [9, 10]. A recurring theme in literature is technical issues, such as system glitches and improper calibration, that affect the overall effectiveness of the simulation [2, 12]. One participant stated, “*We sometimes faced significant difficulties completing assigned tasks due to system glitches*,* making it challenging to meet deadlines effectively.*” Additionally, some students expressed frustration with improper calibration, which affected the overall effectiveness of the simulation. “*VR feels more like a game*,* and the vibrations don’t simulate the actual feel of a handpiece*,” said one participant, making them less confident in their skills. Despite these challenges, VR has proven to be a valuable tool in dental education. It creates practical clinical scenarios and provides force feedback, making training repeatable, reversible, and environmentally friendly without the risk of wasting resources [12, 18, 19]. One participant stated, “*VR allows me to practice without worrying about breaking tools or damaging the manikin. I can practice the same procedure repeatedly until I get it right.*” Additionally, VR was highly valued for helping students understand abstract concepts such as handpiece speed, pressure control, and the differentiation of tooth layers during procedures. One participant stated, “*In VR*,* I can practice handpiece control*,* learn about different caries types*,* and even understand how the tooth layers feel when drilling—things that are difficult to replicate on manikins*.” However, VR’s primary limitation lies in its inability to replicate the sensory experience required to manage real-life clinical procedures. One participant stated, “*While VR can teach me the steps of a procedure*,* it doesn’t provide the sensory feedback needed to manage real-life patient scenarios*.”

Previous studies have demonstrated that virtual reality training can significantly enhance learning outcomes, improve motor skills, minimize procedural errors, and increase the confidence of dental students [10, 20–22]. In this study, the questionnaire assessed preclinical and clinical students’ perceptions and experiences with VR simulation and traditional manikin training, emphasizing their confidence and perceived readiness for clinical practice. We analyzed the data separately for preclinical and clinical groups to explore potential differences. Overall, perceptions of training methods were similar within each group. Notably, the two groups observed a significant difference in the perception of dental manikin training. Preclinical students rated manikin training slightly lower, suggesting that increased clinical exposure leads to a more critical view of this training method. In contrast, both preclinical and clinical students saw VR as similarly useful, suggesting its potential as a versatile tool across all levels of dental education. Both students valued the virtual platform’s visual and tactile experiences. Most students (68.6%) agreed that both training methods are equally effective in consolidating theoretical knowledge, highlighting that the two tools complement each other in reinforcing core learning. Likewise, 69% of students reported improved learning proficiency after using both manikins and VR, suggesting that these methods are valuable supplements to traditional teaching. These outcomes concur with other reports, which have demonstrated that the combined effect of both tools appears to be especially effective in bridging the gap between theoretical knowledge and practical application [6, 20]. 

However, when asked about the realism of clinical scenarios, dental manikins were overwhelmingly preferred (79.9%), with students citing them as a more effective tool for simulating real-world dental procedures (77.3%). This finding is consistent with the primary strength of dental manikins: “*Manikin training provides a more realistic simulation of clinical scenarios and prepares me better for real-life dental procedures*,” one participant stated.

Despite this preference for dental manikins in simulated clinical scenarios, virtual reality was rated as more engaging and motivating, with 58.1% of students finding it better at maintaining their engagement. VR also received high marks for helping students differentiate between the textures and hardness of enamel and dentine (97.4%), demonstrating its value in developing tactile knowledge that is difficult to achieve through working on phantom teeth. Simulating the tactile experience of working with different dental materials can greatly enhance students’ understanding of clinical procedures, making VR an excellent complementary tool for building tactile and sensory awareness [23]. 

Despite these advantages, VR was not seen as a complete replacement for manikin training [3, 8, 18]. Several students preferred an amalgamated approach that leverages the strengths of both methods, with VR being introduced early in the curriculum, helping students visualize procedures and practice basic skills, with manikin training becoming more central as students progress to more complex procedures. This combined approach allows students to develop theoretical understanding and practical skills [1, 20, 24, 25]. One student stated, “*VR might be good for initial exposure*,* but manikin training is still essential for building confidence and skills for real-life patient care*.”

Given the strengths and weaknesses identified in both VR and manikin training, it is clear that an integrated approach would offer the most effective learning experience for dental students. VR can be an excellent tool for reinforcing theoretical knowledge, visualizing procedures, and providing repetitive practice in the early years of dental education. It can also teach fine motor control, such as managing handpiece speed and pressure, and familiarise students with complex concepts that may be difficult to grasp using traditional methods.

However, as students progress in their education, manikin training should continue to play a critical role in developing tactile skills and simulating real-life patient scenarios. Manikins provide the hands-on experience necessary for proficiency in complex clinical procedures, such as cavity preparation, root canal treatment, and rubber dam placement. One participant stated, “*Manikin training should be prioritized for more complex procedures because it provides the hands-on experience and tactile feedback necessary for real-life practice*.”

This study acknowledges several limitations that may affect the interpretation of the findings. As an observational study focused on perceptions rather than experimental outcomes, the lack of a control group makes it difficult to conclude cause and effect. Additionally, the study did not include knowledge acquisition, skill retention, or long-term performance measures, which would have provided a more comprehensive understanding of the training’s effectiveness. Some students also experienced technical glitches and navigation challenges, which could have impacted their overall experience and performance. Moreover, several confounding variables may have influenced the results, including students’ prior experience with technology, varying levels of instructor support during training, individual learning preferences, and differences in academic background and clinical exposure between preclinical and clinical students. The quality and realism of the virtual reality simulators and manikins used in the training might also have affected participants’ experiences and outcomes. Additionally, conducting this study at a single institution is another limitation. These limitations highlight key areas for improvement in future research.

## Conclusion

The current study compares dental students’ perceptions of VR simulation vs. dental manikins as training tools at Ziauddin University. It found that both training methods effectively enhanced learning proficiency but had distinct advantages and limitations. However, VR offers more opportunities for repetitive practice, visualization, and engagement but lacks realism. On the other hand, manikins remain essential for developing the hands-on skills necessary for patient care.

We found complementary strengths in both tools, and our study highlights the importance of integrating VR and manikin training in dental curricula to offer a comprehensive educational experience.

We suggest future research on integrating VR technologies into dental programs, particularly in resource-limited environments, to evaluate their cost-effectiveness and long-term clinical performance. Strategies to address VR’s lack of realism also need to be explored.

## Electronic supplementary material

Below is the link to the electronic supplementary material.

Supplementary Material 1

## Data Availability

The data that support the findings of this study are available from the authors upon reasonable request.

## Abbreviations

VR: Virtual reality
ZU: Ziauddin University
BDS: Bachelor of Dental Surgery
