# Supplementary material for: Exploring the frontier of dental education: a cross-sectional study of VR simulation and manikin-based training at Ziauddin university
Source: BMC Med Educ. 2025 May 12;25:695. doi: 10.1186/s12909-025-07221-8 (PMC12070776; doi:10.1186/s12909-025-07221-8)
Supplement: Supplementary file 1 — Supplementary Material 1 [file 12909_2025_7221_MOESM1_ESM.pdf]

Exploring the Frontier of Dental Education: A Cross-Sectional study comparing the perception of students on VR Simulation and Traditional Dental Manikins as preclinical teaching tools at Ziauddin University.

SECTION 1: PERSONAL DATA

Gender _________
Age _________
Year of study in dental program _________

SECTION 2: EXPERIENCE WITH DENTAL MANIKINS

No	Component	Completely agree	Agree	Neutral	Disagree	Completely disagree
1	Using this method is easy for me					
2	This method is highly reliable					
3	Using this method increases accuracy					
4	Using this method promotes comprehension					
5	Time can be managed better by this method					
6	Learning is more enjoyable with this method					
7	I achieved the course objectives with this method					
8	I felt at ease by using this method					
9	I am completely satisfied with this method					

SECTION 3: EXPERIENCE WITH VIRTUAL REALITY TRAINING

No	Component	Completely agree	Agree	Neutral	Disagree	Completely disagree
1	Using this method is easy for me					
2	This method is highly reliable					
3	Using this method increases the accuracy					
4	Using this method promotes comprehension					
5	Time can be managed better by this method					
6	Learning is more enjoyable with this method					
7	I achieved the course objectives with this method					
8	I felt at ease by using this method					
9	I am completely satisfied with this method					

SECTION 4: COMPARATIVE QUESTIONS

1. Which method helps to consolidate theoretical knowledge?	VR	Dental Manikin	Both equally	Neither
2. Which training method improves learning proficiency?				
3. Which method offers a more realistic simulation of clinical scenarios?				
4. Which training method better prepares you for real-life dental procedures?				
5. Which training method do you prefer for learning new clinical skills?				
6. Which method offers a more comprehensive understanding of dental procedures?				
7. After using both VR and dental manikins, which method left you feeling more confident about performing real-life dental procedures?				
8. Comparing VR versus dental manikin experience which method keeps you more engaged and motivated?				
9. Reflecting on your experiences, which training method do you feel more eager to use?				

10. Which helps me differentiate between the texture and hardness of enamel and dentine?				

SECTION 5: FEEDBACK

1. Any limitations or challenges you have encountered with using dental manikins for clinical skills training?

   ____

   ____

2. Any limitations or challenges you have encountered with using VR for clinical skills training?

   ____

   ____

3. For which specific dental skills do you find VR training more effective than manikin training?

   ____

   ____

4. Are there any specific dental skills or procedures where you found manikin training superior to VR?

   ____

   ____

5. Given the choice, how would you like to see the balance between VR and manikin training in future dental curricula?

   ____

   ____
